# A Novel Case of Idiopathic Restrictive Cardiomyopathy

**DOI:** 10.7759/cureus.7212

**Published:** 2020-03-08

**Authors:** Talha Ahmed, Ayesha Safdar, Gautam Ramani

**Affiliations:** 1 Internal Medicine, University of Maryland, Baltimore, USA; 2 Internal Medicine, Army Medical College, Rawalpindi, PAK; 3 Cardiology, University of Maryland Medical Center, Baltimore, USA

**Keywords:** restrictive cardiomyopathy, idiopathic, orthotopic liver transplant, paracentesis, dialysis, cardiac biopsy, idiopathic restrictive cardiomyopathy, liver transplant, renal dialysis, biopsy

## Abstract

Restrictive cardiomyopathy (CM) usually develops and progresses slowly, over a course of years. The rapid development of idiopathic restrictive CM immediately following a liver transplant is unusual. We describe the case of a patient who developed idiopathic restrictive CM fairly rapidly following a liver transplant. It progressed within a few months to the point where the patient required scheduled paracenteses and dialysis. The morphological definition of restrictive CM consists of bi-atrial dilation with non-dilated and non-hypertrophic ventricles. A cardiac biopsy may be needed when the underlying cause is not evident. When a cardiac biopsy is not able to identify a specific cause, then the word "idiopathic" is used to describe the CM.

## Introduction

Most restrictive cardiomyopathies (RCMs) are due to the infiltration of abnormal substances between myocytes, storage of abnormal metabolic products within myocytes, or fibrotic injury [1]. RCM usually develops and progresses slowly, over a course of years [2]. The rapid development of idiopathic RCM immediately following a liver transplant is unusual and has not been described heretofore in the past.

## Case presentation

A 62-year-old male with a known history of cirrhosis secondary to alcohol use and untreated hepatitis C was scheduled for orthotropic liver transplant (OLT). As a part of pre-operative optimization, he was found to have severe aortic stenosis and underwent trans-catheter aortic valve replacement followed by an uneventful liver transplant. Routine subsequent immunosuppression included tacrolimus and mycophenolate.

**Figure 1:**
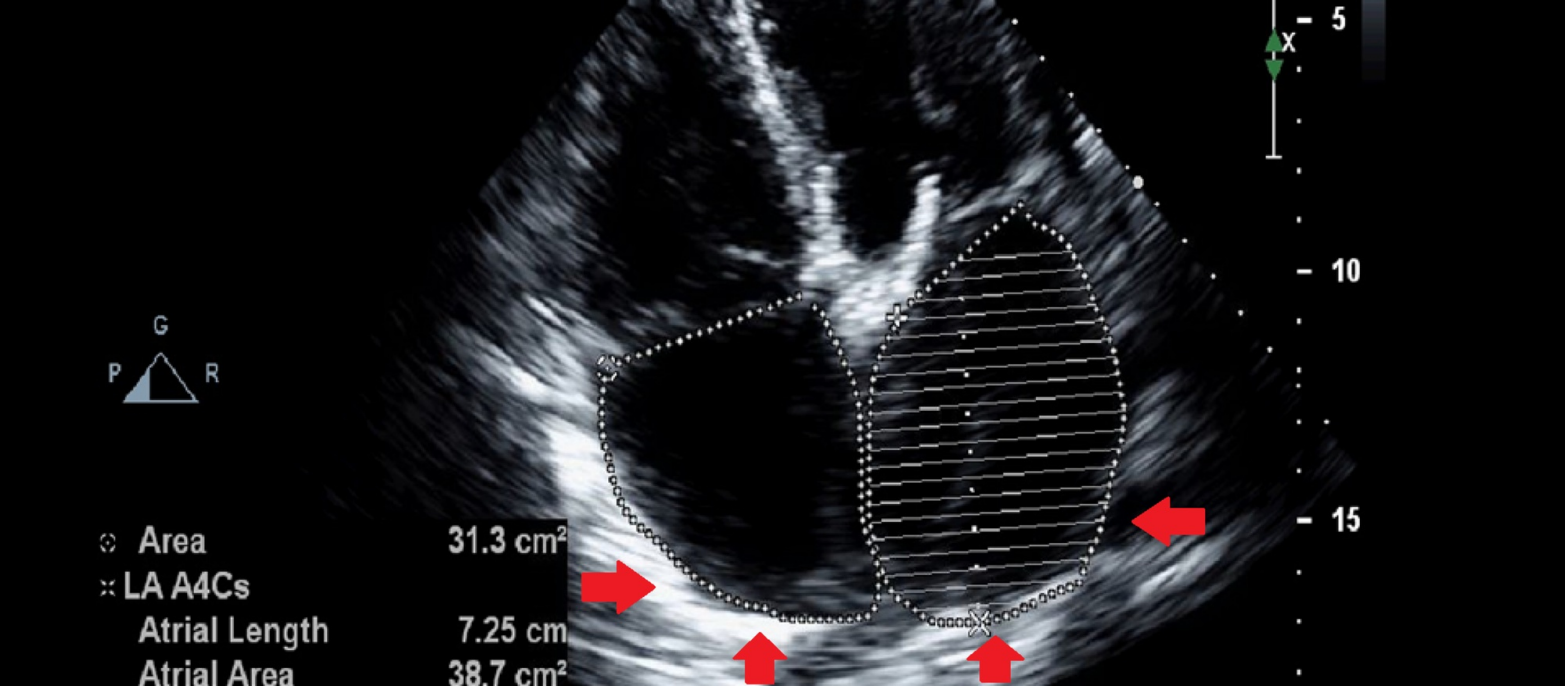
Transthoracic echocardiogram with an apical four-chamber view showing bi-atrial dilation

**Figure 2:**
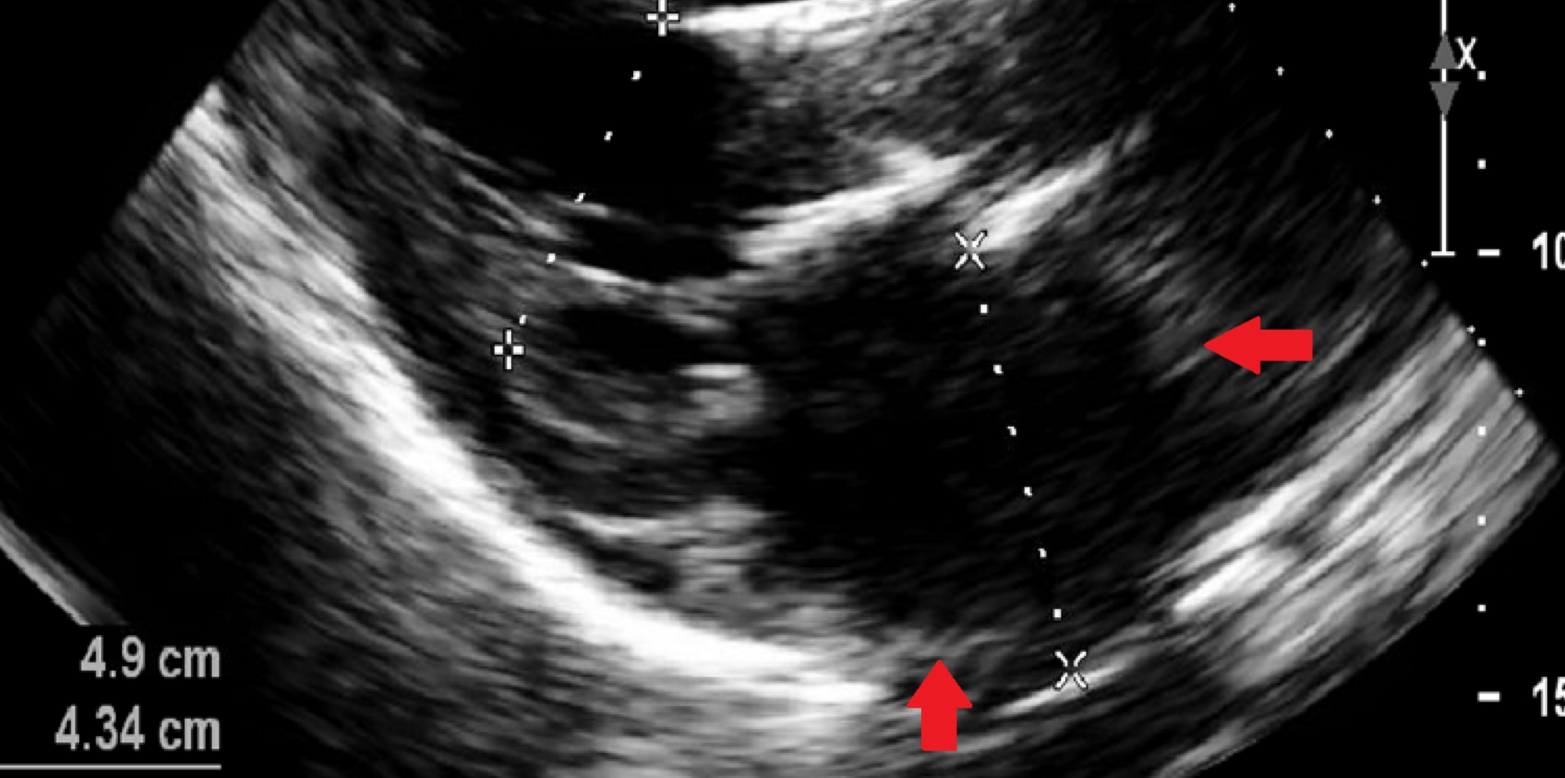
Transthoracic echocardiogram with a parasternal long-axis view showing dilated left atrium

A few months later, the patient started having exertional dyspnea with ascites and lower extremity edema. NT-proBNP (N-terminal pro-b-type natriuretic peptide) was mildly elevated at 400 pg/mL (normally it is less than 125 pg/mL for patients between 0 and 74 years of age). Echocardiogram showed bi-atrial dilation, with non-dilated, non-hypertrophied ventricles and worsening bi-ventricular systolic function with elevated right atrial and pulmonary artery pressures, consistent with an RCM (Figures 1, 2). The phenomenon of septal bounce was not present on the echocardiogram.

Right heart catheterization (RHC) showed elevated right- and left-sided pressures with moderate pulmonary hypertension (PH). A careful review did not reveal any history of pericarditis. New-onset ascites was considered to be cardiac in etiology as liver function tests, abdominal ultrasound, and liver biopsy all demonstrated normal allograft function. Left ventricular function showed improvement with medication optimization, but the PH and right ventricular function continued to worsen, resulting in diuretic refractory ascites that mandated multiple sequential paracenteses. Renal function also declined, necessitating hemodialysis for fluid removal. An outpatient RHC with constriction/restriction study was non-diagnostic. An endomyocardial biopsy was performed, which did not reveal an infiltrative disease and demonstrated patchy endocardial and interstitial fibrosis with compensatory myofibril hypertrophy without myofiber disarray, hence favoring the diagnosis of 'idiopathic restrictive cardiomyopathy'. Cardiac MRI was deferred due to renal insufficiency, whereas nuclear imaging such as cardiac single-photon emission computed tomography could not be performed due to a lack of institutional availability.

## Discussion

Most RCMs are due to the infiltration of abnormal substances between myocytes, storage of abnormal metabolic products within myocytes, or fibrotic injury. In idiopathic RCM, however, no definitive cause can be elucidated on cardiac biopsy [3]. RCM usually develops and progresses slowly, over a course of years. The rapid development of idiopathic RCM immediately following a liver transplant is unusual and has not been described heretofore in the past [4-5].

Differential diagnosis of RCM due to myocardial causes are divided into non-infiltrative pattern, which includes familial CM, hypertrophic CM, scleroderma, pseudoxanthoma elasticum, diabetic CM, and idiopathic CM. The infiltrative pattern of RCM includes amyloidosis, sarcoidosis, Gaucher’s disease, Hurler’s disease, and fatty infiltration. Storage diseases such as hemochromatosis, Fabry’s disease, and glycogen storage diseases also are myocardial causes of RCM. Endomyocardial etiologies include hyper-eosinophilic syndrome, carcinoid heart, metastatic cancer, radiation, and drugs (anthracycline, serotonin, methysergide, ergotamine, busulfan, and mercurial agents) [6-7].

The morphological definition of idiopathic RCM is based on echocardiographic features demonstrating non-dilated, non-hypertrophied ventricles, with marked bi-atrial enlargement in the absence of ischemic, valvular, hypertensive, congenital, inflammatory, or infiltrative heart diseases. Elevated RA pressures and PH are usually present [8]. In idiopathic RCM, as demonstrated in our case, myocardial biopsy typically demonstrates patchy endocardial and interstitial fibrosis with compensatory myofibril hypertrophy without myofiber disarray, or findings suggestive of a specific infiltrative heart muscle disease [9-10].

This is a rare case of idiopathic RCM occurring less than one year after a liver transplant that was rapidly progressive, with the development of refractory ascites and renal failure necessitating dialysis. A similar phenomenon has not been described in the past. Though the underlying mechanism is not known, a relationship with OLT is suspected.

## Conclusions

We conclude that idiopathic RCM can rarely develop following a liver transplant and can progress rapidly over the course of months, leading to refractory heart failure symptoms and end-organ damage. This should be considered as a differential diagnosis in patients who develop worsening ascites and edema with normally functioning liver allograft. Cardiac biopsy is usually non-diagnostic and reveals focal interstitial fibrosis with mild myocyte hypertrophy without specific evidence of any infiltrative disease. Transthoracic echocardiographic findings of bi-atrial dilation with non-dilated and non-hypertrophied ventricles usually suggest the diagnosis of idiopathic RCM in these cases.
